# Therapeutic targeting of mineralocorticoid receptors in pulmonary hypertension: Insights from basic research

**DOI:** 10.3389/fcvm.2023.1118516

**Published:** 2023-01-30

**Authors:** Argen Mamazhakypov, Achim Lother

**Affiliations:** ^1^Institute of Experimental and Clinical Pharmacology and Toxicology, Faculty of Medicine, University of Freiburg, Freiburg im Breisgau, Germany; ^2^Faculty of Medicine, Interdisciplinary Medical Intensive Care, Medical Center, University of Freiburg, Freiburg im Breisgau, Germany

**Keywords:** pulmonary hypertension, aldosterone, finerenone, spironolactone, mineralocorticoid receptors, eplerenone, right heart failure

## Abstract

Pulmonary hypertension (PH) is characterized by pulmonary vascular remodeling and associated with adverse outcomes. In patients with PH, plasma aldosterone levels are elevated, suggesting that aldosterone and its receptor, the mineralocorticoid receptor (MR), play an important role in the pathophysiology of PH. The MR plays a crucial role in adverse cardiac remodeling in left heart failure. A series of experimental studies from the past few years indicate that MR activation promotes adverse cellular processes that lead to pulmonary vascular remodeling, including endothelial cell apoptosis, smooth muscle cell (SMC) proliferation, pulmonary vascular fibrosis, and inflammation. Accordingly, *in vivo* studies have demonstrated that pharmacological inhibition or cell-specific deletion of the MR can prevent disease progression and partially reverse established PH phenotypes. In this review, we summarize recent advances in MR signaling in pulmonary vascular remodeling based on preclinical research and discuss the potential, but also the challenges, in bringing MR antagonists (MRAs) into clinical application.

## Introduction

Pulmonary hypertension (PH) is characterized by increased muscularization and thickening of the small pulmonary arteries (PAs), resulting in progressive elevation of pulmonary vascular resistance (PVR) and PA pressure (PAP) (1). With disease progression, increased right ventricular (RV) afterload leads to RV dysfunction and failure, resulting in markedly reduced functional capacity, quality of life, and life expectancy (2). PH is defined as a mean PAP (mPAP) of more than or equal to 20 mmHg and PVR ≥ 3 Wood Units (WU) for pre-capillary forms of PH, measured by right heart catheterization (3–5). PH combines heterogeneous pulmonary vascular conditions, classified into five groups as follows: Group 1—pulmonary arterial hypertension (PAH), including idiopathic, heritable, and drug/toxin-induced PH; Group 2—PH due to left heart disease; Group 3—PH due to lung disease and/or chronic hypoxia; Group 4—PH due to chronic thromboembolism; and Group 5—PH with unclear multi-factorial mechanisms.

The pathobiology of pulmonary vascular remodeling is characterized by PA endothelial cell (PAEC) dysfunction and apoptosis with the subsequent reactive proliferation of PA smooth muscle cells (PASMCs), increased extracellular matrix (ECM) deposition, and inflammatory/immune cell infiltration of the pulmonary vascular wall (1). Despite several decades of research in this field, disease development and progression mechanisms remain incompletely defined (6). Alterations of several signaling pathways have been shown to participate in the pathogenesis of pulmonary vascular remodeling. This has led to the development and approval of therapies that primarily target calcium channels, prostaglandin receptors, endothelin receptors (ETs), phosphodiesterases, and soluble guanylate cyclase in PAH (5). However, these therapies provide only partial improvements in hemodynamics and outcome for PAH patients suggesting there are additional dysregulated signaling pathways contributing to the disease pathogenesis. In addition, available therapies are largely restricted to Group 1 PH patients (5) (except for riociguat in CTEPH) but not efficient or even detrimental in patients with other PH classes.

Therefore, a number of preclinical and early clinical trials have been conducted in order to test the ability of pharmacological agents targeting other signaling pathways to improve the pathophysiology of PH and patient outcomes (7). Among others, augmented activation of the mineralocorticoid receptor (MR), an aldosterone receptor belonging to the nuclear transcription receptor superfamily, has emerged as one of the underlying mechanisms driving disease development and progression in PH (8). Aldosterone is a critical effector hormone of the renin-angiotensin-aldosterone system (RAAS), which plays an important role in the regulation of normal cardiovascular homeostasis and the pathogenesis of diverse cardiovascular diseases. Activation of MR in cardiomyocytes, endothelial cells, vascular SMCs, or myeloid cells induces inflammation and adverse remodeling of the heart and the vascular system (9, 10). MR antagonists (MRAs) such as spironolactone and eplerenone are considered standard in left heart failure management and associated with significantly improved outcomes (10).

During the past decade, a series of experimental studies investigated the role of aldosterone and MR in pulmonary vascular remodeling and a potential benefit of MRAs for PH patients. In this review, we discuss mechanisms of MR signaling in pulmonary vascular cells with a focus on recent findings from genetically engineered animal models. We compare the impact of pharmacological MR blockade in different PH animal models and the association of aldosterone with PH patients’ phenotypes and outcomes. Lastly, we discuss the translational potential of MRAs toward clinical application in PH, as well as new research initiatives that may lead to a better understanding of MR in PH and further developments.

### Mechanisms of aldosterone and mineralocorticoid receptor signaling

The mineralocorticoid aldosterone is primarily synthesized in adrenocortical cells of the zona glomerulosa of the adrenal cortex and functions as one of the effector hormones in the RAAS. The classical role of aldosterone is to control salt and water balance *via* binding to the MR in kidney epithelial cells, however, MR is widely expressed in extrarenal tissues (11).

The MR belongs to the family of nuclear receptors of ligand-dependent transcription factors (12). Unbound MR is together with its chaperone proteins located in the cytosol. Ligand binding induces a conformational change of the MR, dissociation from the chaperone proteins, dimerization, and translocation into the nucleus to induce the expression of target genes (13). Besides aldosterone, cortisol acts as a ligand at the MR with similar degree of affinity as aldosterone (11). Given the high concentration of cortisol in tissues and circulation, aldosterone binding to the MR requires the expression of 11β-hydroxysteroid dehydrogenase 2 (HSD11B2), which inactivates cortisol into cortisone, which has low affinity for the MR (11, 14). In addition to regulating gene expression, MR also can promote non-genomic effects by modulating several other signaling pathways, including different membrane receptors (13).

### Pharmacology of the mineralocorticoid receptor

Mineralocorticoid receptor antagonists comprise a group of pharmacological agents that antagonize the action of aldosterone at the MR. Several MRAs including spironolactone, eplerenone, and finerenone have been studied for their effects in the experimental models of PH (Tables 1, 2). Despite the similar mechanisms of action of those agents, they may substantially differ between each other in term of their pharmacological properties, which may eventually account for the variability of the observed effects in humans and animal models. For example, spironolactone exhibits binding affinity not only to the MR but also to androgen and progesterone receptors, causing undesirable effects such as painful gynecomastia (15). This was improved with eplerenone, showing an improved selectivity for MR and less side effects due to unspecific binding to androgen and progesterone receptors compared to spironolactone (15). In addition, eplerenone exhibits lower plasma protein binding and has a shorter plasma half-life compared with spironolactone (16). Both, spironolactone and eplerenone are based on a steroidal backbone. More recently, non-steroidal MRAs such as finerenone or esaxerenone have been developed, which have specific pharmacological features that are distinct from steroidal MRAs. So far, finerenone has been tested in experimental PH. Finerenone is a dihydronaphthyridine-based compound with high selectivity for the MR over all other steroid hormone receptors and high binding affinity (17). Finerenone is excreted to a minor degree (<1%) by the kidney, has a short plasma half-life (2–3 h), also in patients with renal failure, and no active metabolites have been identified (18). These pharmacological features of finerenone may contribute to a significantly smaller increases in serum potassium levels and lower incidences of hyperkalemia compared to the steroidal MRA spironolactone (19). Collectively, the pharmacological characteristics of the MRAs outlined above should be carefully considered when interpreting and comparing the observed effects and side effects of these drugs. The pharmacological characteristics of the different MRAs outlined above should be taken into account when evaluating the potential utility of these compounds in preclinical and clinical studies of PH.

**TABLE 1:** Summary of studies evaluating the preventive application of mineralocorticoid receptor antagonists (MRAs) in rodent models of pulmonary hypertension (PH).

Agent	PH model	Agent details	Pulmonary hemodynamics		Vascular remodeling (histo)	RV remodeling (echo)	RV remodeling (ex-vivo, histo)	RV function (echo)	References
			**Invasive**	**Non-invasive**					
Spironolactone	Monocrotaline rats (3 weeks)	40 mg/kg/day (implanted pellet) (3 weeks)	↓ RVSP	NA	↓ PA muscularization	NA	↔ RV/(LV+S), ↓ RV myocyte, ↔ RV fibrosis	NA	(44)
Spironolactone	Monocrotaline rats (3 weeks)	25 mg/kg/day (drinking water) (3 weeks)	↓ PASP	NA	↓ PA muscularization, ↓ Vessel wall thickness	NA	↓ RV/(LV+S)	NA	(46)
Spironolactone	Monocrotaline rats (25 days)	25 mg/kg/day (drinking water) (25 days)	↓ PASP	↓ PAAT	↓ PA muscularization, ↓ PA fibrosis	↓ RVWT	NA	NA	(27)
Spironolactone	Hypoxia-sugen mice (4 weeks)	15 mg/kg/day (implanted pellet)	↓ RVSP	NA	↓ PA muscularization	NA	↔ RV/(LV+S), ↓ RV myocyte, ↓ RV fibrosis	NA	(47)
Spironolactone	Hypoxia mice (5 weeks)	15 mg/kg/day (implanted pellet) (5 weeks)	↓ RVSP	NA	↓ PA muscularization	NA	↔ RV/(LV+S), ↓ RV fibrosis, ↔ RV myocyte	NA	(44)
Eplerenone	Hypoxia mice (6 weeks)	200 mg/kg/d (chow) (5 weeks)	NA	↑ PAAT/PAET	↓ PA vessel thickness	↓ RVID/LVID	↓ RV myocyte	↑ TAPSE	(52)
Eplerenone	Hypoxia-sugen mice (3 weeks)	200 mg/kg/d (chow) (3 weeks)	↓ RVSP	NA	↓ PA muscularization, ↓ Vessel wall thickness	NA	↓ RV/BW	NA	(51)
Spironolactone	Monocrotaline rats (25 days)	25 mg/kg/day (drinking water) (25 days)	NA	NA	↓ PA wall thickness	NA	NA	NA	(54)

RVSP, right ventricular systolic pressure; mPAP, mean pulmonary artery pressure; PSAP, pulmonary artery systolic pressure; BW, body weight; RV, right ventricle; LV, left ventricle; S, septum; PA, pulmonary artery; TAPSE, tricuspid annular plane systolic excursion; RVWT, right ventricular wall thickness; RVID, right ventricular diameter at end-diastole; LVID, left ventricular internal diameter; NA, not applicable. ↔, Measured parameter in the treatment did not change significantly compared to placebo group; ↑, measured parameter in the treatment significantly increased compared to placebo group; ↓, measured parameter in the treatment significantly decreased compared to placebo group.

**TABLE 2:** Summary of studies evaluating the therapeutic application of mineralocorticoid receptor antagonists (MRAs) in rodent models of pulmonary hypertension (PH).

Agent	PH model	Agent details	Pulmonary hemodynamics		Vascular remodeling (histo)	RV remodeling (echo)	RV remodeling (ex-vivo, histo)	RV function (echo)	References
			**Invasive**	**Non-invasive**					
Spironolactone	Monocrotaline rats (25 days)	25 mg/kg/day (drinking water) (10 days)	↓ RVSP	NA	NA	NA	NA	NA	(27)
Spironolactone	Monocrotaline rats (5 weeks)	40 mg/kg/day (implanted pellet) (2 weeks)	↓ RVSP	NA	↓ PA muscularization	NA	↔ RV/(LV+S), ↓ RV myocyte, ↔ RV fibrosis	NA	(44)
Spironolactone	Hypoxia-sugen rats (6 weeks)	25 mg/kg/day (drinking water) (4 weeks)	↓ PASP	NA	NA	NA	↓ RV/LV	NA	(26)
Spironolactone	Hypoxia-sugen rats (6 weeks)	25 mg/kg/day (drinking water) (3 weeks)	↓ PASP	NA	NA	NA	↓ RV/(LV+S)	NA	(46)
Spironolactone	Hypoxia-sugen rats (10 weeks)	40 mg/kg/day (chow) (5 weeks)	↔ RVSP	NA	↔ PA wall thickness	↔ LV ECI (MRI), ↔ RVEDV/LVEDV (MRI)	↔ RV fibrosis	↔ RV EF (MRI)	(53)
Eplerenone	Hypoxia-sugen rats (10 weeks)	100 mg/kg/day (chow) (5 weeks)	↔ RVSP	NA	↔ PA wall thickness	↓ LV ECI (MRI), ↓ RVEDV/LVEDV (MRI)	↔ RV fibrosis	↔ RV EF (MRI)	(53)
Eplerenone	Pulmonary artery banding mice (3 weeks)	200 mg/kg/d (chow) (3 weeks)	↔ RVSP	NA	NA	↔ RVID	↔ RV/BW, ↔ RV fibrosis, ↔ RV cardiomyocyte	↔ TAPSE	(51)
Finerenone	Monocrotaline rats (4 weeks)	1 mg/kg/day (per os) (2 weeks)	↓ mPAP	↑ PAAT/PAET	↓ PA muscularization, ↓ PA wall thickness	NA	↓ RV/(LV+S), ↓ RV fibrosis	NA	(43)
Finerenone	Hypoxia-sugen rats (8 weeks)	1 mg/kg/day (per os) (3 weeks)	↓ mPAP	↑ PAAT/PAET	↓ PA muscularization, ↓ PA wall thickness	NA	↓ RV/(LV+S), ↓ RV fibrosis	NA	(43)

RVSP, right ventricular systolic pressure; mPAP, mean pulmonary artery pressure; PSAP, pulmonary artery systolic pressure; BW, body weight; RV, right ventricle; LV, left ventricle; S, septum; PA, pulmonary artery; TAPSE, tricuspid annular plane systolic excursion; RVWT, right ventricular wall thickness; RVID, right ventricular diameter at end-diastole; LVID, left ventricular internal diameter; NA, not applicable. ↔, Measured parameter in the treatment did not change significantly compared to placebo group; ↑, measured parameter in the treatment significantly increased compared to placebo group; ↓, measured parameter in the treatment significantly decreased compared to placebo group.

## Effects of aldosterone and MR activation on the course and severity of PH

**FIGURE 1:**
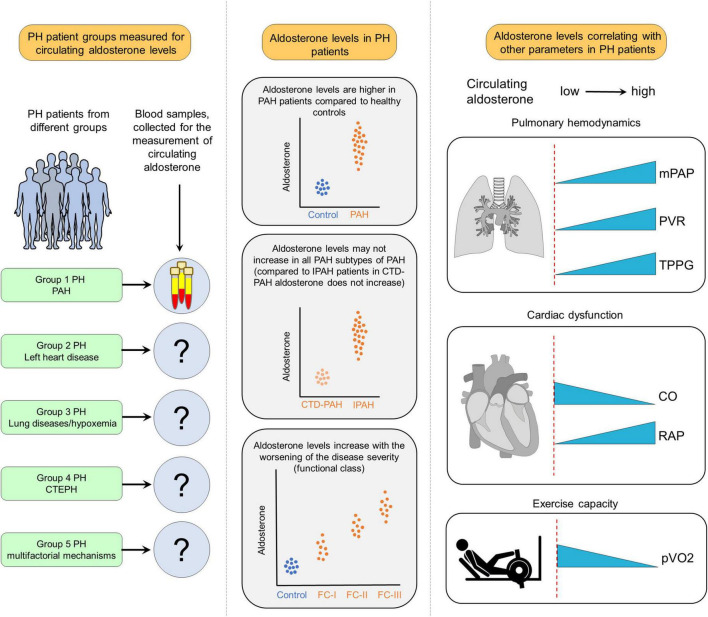
Clinical implications of increased aldosterone levels in pulmonary hypertension (PH). In available studies, aldosterone levels have been measured mostly in Group 1 PH also referred to as pulmonary arterial hypertension (PAH), while in other forms of PH, circulating levels of aldosterone remained neglected. The results of those studies revealed that aldosterone levels are increased in PAH patients compared to healthy controls. In addition, there are differences in PAH subtypes regarding aldosterone levels. For example, compared to idiopathic PAH (IPAH), circulating aldosterone levels are not increased in PAH patients due to connective tissue diseases (CTD-PAH). Moreover, in PAH patients, aldosterone levels are increased along with worsening the disease severity. Furthermore, aldosterone levels are correlated with several hemodynamic and functional parameters like mean PA pressure (mPAP), pulmonary vascular resistance (PVR), transpulmonary pressure gradient (TPPG), cardiac output (CO), right atrial pressure (RAP), and decreased maximal oxygen uptake (pVO2).

Patients with PH with no evidence of left heart dysfunction display elevated aldosterone levels, which are associated with a wide range of clinical and hemodynamic indices (20, 21) (Figure 1). For example, in patients with PAH aldosterone levels showed positive correlations with PVR, mPAP, transpulmonary pressure gradient, and WHO functional class (22, 23) but negative correlations with cardiac output (CO) (22). Furthermore, aldosterone level in PAH patients may differ depending on the underlying etiology. For example, in contrast to idiopathic PAH (IPAH) patients, circulating aldosterone levels are not increased in PAH patients associated with connective tissue diseases (PAH-CTD) (23). Taken together, these studies indicate that circulating aldosterone levels are increased in Group 1 PH and its increase may be associated with adverse functional and hemodynamic alterations. Available data suggest that both, adrenal and extra-adrenal sources may contribute to overall circulating levels of aldosterone in PAH. For example, in PH due to heart failure with preserved ejection fraction (HFpEF), transpulmonary aldosterone levels are increased indicating that pulmonary synthesis of aldosterone (24). In more advanced disease conditions, altered hemodynamics may contribute to an exaggerated increase of circulating aldosterone. For example, decompensated stage of PH with pronounced RV failure may lead to the release of renin secondary to chronically reduced kidney perfusion resulting in chronically activated RAAS (25).

## Local and systemic aldosterone synthesis in pulmonary hypertension

The contribution of local aldosterone production in lung tissue during PH has been assessed in experimental models. In hypoxia-sugen rat (26) and monocrotaline rat (27) models, PH development is associated with not only increased circulating aldosterone but also lung tissue aldosterone content. Two rate-limiting enzymes catalyze the formation of aldosterone from cholesterol: steroidogenic acute regulatory protein (StAR), which transports cholesterol to the inner mitochondrial membrane, and aldosterone synthase (CYP11B2), which converts 11-deoxycorticosterone into aldosterone. In addition, aldosterone in PAECs may also be synthesized from other metabolic intermediates such as pregnenolone, rather than from cholesterol (28), which can explain the differences in the expression profiles of enzymes responsible for aldosterone synthesis in PAECs compared to cells of the adrenal cortex (28). *In vitro* studies have shown that stimulation of PAECs with ET-1 induces the binding of the steroidogenic transcription factors such as steroidogenic factor-1 (SF-1) and peroxisome proliferator-activated receptor-γ coactivator-1α (PGC-1α) to the CYP11B2 promoter, leading to CYP11B2 expression and aldosterone synthesis (27). Similarly, hypoxia also promotes PAEC aldosterone synthesis by increasing StAR expression *via* hypoxia-induced c-Fos/c-Jun binding to the StAR promoter (26). Moreover, angiotensin-II (Ang-II) also has been shown to induce aldosterone synthesis in PAECs (27). Cumulatively, these studies indicate that various factors, including ET-1, Ang-II, and hypoxia, which have been known to modulate pulmonary vascular remodeling, may also directly stimulate *de novo* aldosterone synthesis in the pulmonary vasculature.

## Aldosterone–MR signaling in pulmonary vascular cells

Mineralocorticoid receptor is expressed in PAECs and PASMCs and MR activation induces various cellular processes that contribute to pulmonary vascular remodeling in PH: in healthy PAECs, nitric oxide (NO) is produced by endothelial nitric oxide synthase (eNOS) and diffuses to the underlying PASMCs, where it promotes pulmonary vascular dilation (29). In addition, NO also acts locally on PAECs to prevent inflammation and thrombosis. In PH, this system becomes dysregulated and is considered a key driver of pulmonary vascular remodeling (30). MR activation by aldosterone as observed in PH disturbs NO signaling by promoting reactive oxygen species (ROS) production in pulmonary vascular cells. Aldosterone activates NADPH oxidase 4 in PAECs to generate excessive ROS (31, 32), which in turn leads to NEDD9 oxidation, disabling its association with SMAD3, and resulting in NEDD9 nuclear translocation (31). In the nucleus of PAECs, NEDD9 together with NKX2 activates the COL3A1 gene promoter (31). In addition, aldosterone-mediated expression of profibrotic factors including connective tissue growth factor (CTGF), collagen 1, matrix metalloprotease 2 (MMP2), and MMP9 in PAECs (26, 31) has been shown, which is considered to ultimately result in excessive collagen deposition in the pulmonary vasculature.

Hydrogen peroxide (H_2_O_2_) generation in PAECs due to aldosterone-mediated NADPH oxidase-4–activity induces a sulfenic post-translational modification of the ET type-B (ET-B), resulting in the blockade of ET-B signaling pathway and impaired NO synthesis and bioavailability in the pulmonary vasculature (27). It is interesting to note that in the systemic vasculature, MR signaling also plays a role in promoting ROS formation and dampening NO signaling that is EC-specific. MR deletion in ECs prevents the aldosterone-induced increase in superoxide (O_2_^–^) production (33) and results in enhanced eNOS activity and improved endothelial function (34). Consequently, MR-mediated decrease in NO bioavailability within the pulmonary vasculature results in PASMC contractility, proliferation, and ECM synthesis (27), which are the main characteristics of the pathobiology of PH. Taken together, impaired NO signaling and excessive ROS formation in PH is considered one of the pathological mechanisms underlying PH development in response to excessive aldosterone.

**FIGURE 2:**
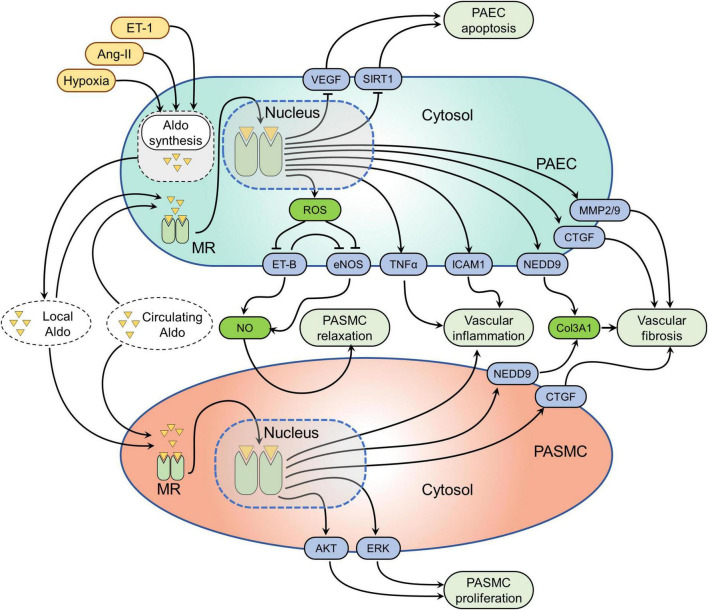
Mineralocorticoid receptor (MR)-mediated signaling pathways in pulmonary vascular cells. Several factors such as hypoxia, angiotensin-II (Ang-II), and endothelin-1 (ET-1) have been shown to induce aldosterone synthesis in pulmonary artery endothelial cells (PAECs). Both locally synthesized aldosterone and circulating aldosterone can activate MR in PAECs and PA smooth muscle cells (PASMCs). Activation of the MR alters several signaling pathways in pulmonary vascular cells. The effected pathways in PAECs are the following: (1) MR-induced reactive oxygen species (ROS) formation, which in turn inhibits endothelial nitric oxide synthesis (eNOS) and endothelin receptor B (ET-B) resulting in decreased nitric oxide (NO) formation. The resulting effect of decreased NO bioavailability is increased pulmonary vascular SMC (PASMC) contractility; (2) MR activation inhibits VEGF and sirtuin 1 (SIRT1) resulting in PAEC apoptosis; (3) MR leads to the overexpression neural precursor cell expressed developmentally down-regulated protein 9 (NEDD9), connective tissue growth factor (CTGF), matrix metalloproteinase-2 and 9 (MMP2/9) causing pulmonary vascular fibrosis; (4) MR also induces tumor necrosis factor alpha (TNFα) and intercellular adhesion molecule-1 (ICAM-1) overexpression, resulting in pulmonary vascular inflammation. While the MR effected pathways in PASMCs include: (1) MR activation leads to pulmonary vascular inflammation through unknown mechanisms; (2) MR induced neural precursor cell expressed developmentally down-regulated protein 9 (NEDD9) and CTGF activation cause pulmonary vascular fibrosis; (3) while MR-mediated activation of protein kinase B (AKT) and extracellular signal-regulated kinase (ERK) signaling pathways cause increased PASMC proliferation.

Activated aldosterone-MR signaling has been shown to promote PAEC senescence and inhibit cell proliferation by down-regulating SIRT1 with consequent p53 and p21 up-regulation (35). Excessive EC apoptosis and pulmonary vascular remodeling due to secretin deficiency are at least partially mediated by aldosterone-mediated down-regulation of vascular endothelial growth factor (VEGF) (36). MR activation is a well-known driver of vascular inflammation (37, 38) and this seems to apply to the pulmonary vasculature as well. In PAECs, MR promotes leukocyte adhesion to ECs *via* up-regulating intercellular adhesion molecule-1 (ICAM-1) expression (39). Similarly, aldosterone-MR signaling also facilitates tumor necrosis factor alpha (TNFα)-induced proinflammatory gene expression in PAECs, which can be effectively prevented by the application of spironolactone (40). In addition, aldosterone may promote pulmonary vascular remodeling by inducing endothelial-to-mesenchymal transition (EndoMT), as it has been demonstrated that MR activation promotes cardiac and renal fibrosis *via* activating EndoMT (41), which is also involved in the pathogenesis of PH (42). However, the direct effects of aldosterone mediated EndoMT in the pathogenesis of PH has not been studied and should be investigated in the future. Finally, MR may interfere with VEGF signaling in PAECs, however, this remains to be confirmed. Taken together, MR signaling activation in PAECs alters diverse cellular processes including NO signaling, ROS formation, cellular apoptosis, ECM synthesis, and inflammation which in crosstalk with PASMCs, fibroblasts, and immune cells promotes pulmonary vascular remodeling and PH development (Figure 2).

Beside indirect signaling *via* MR in PAECs, it has been shown that MR activation in PASMCs is involved in PH progression. A recent study has provided direct experimental evidence that MR is overexpressed in PASMCs of the remodeled PAs in patients with PAH as well as in monocrotaline and hypoxia-sugen rats with established PH (43). *In vitro*, aldosterone activates MR in PASMCs in a dose-dependent manner without affecting its expression level (44). The main effect of activated aldosterone-MR signaling in PASMCs is an augmented cell proliferation (44, 45), which can be prevented by MRAs (44, 45) or MR-directed siRNAs (43). Aldosterone has been shown to modulate various cellular signaling pathways in PASMCs to promote cell proliferation and survival (44). For example, in PASMC, aldosterone activates the Akt signaling pathway, which in turn induces mTOR signaling pathway activation resulting in cell survival and proliferation (46). In addition, aldosterone has been shown to promote PASMC proliferation *via* the ERK signaling pathway because of BMP2/7-mediated MR up-regulation (45). Similarly, aldosterone also promotes PASMC viability *via* up-regulating aquaporin and b-catenin (35). Moreover, aldosterone prevents oxidative stress-induced PASMC apoptosis (35). In addition to increased cell proliferation, aldosterone also promotes the profibrotic phenotype of PASMCs. For example, hypoxia-exposed PAECs promote CTGF up-regulation in PASMCs *via* aldosterone-mediated mechanisms (26). As outlined above, aldosterone-induced NEDD9 up-regulation in PAECs can cause exosome-mediated NEDD9 activation in PASMCs and collagen three up-regulation (31). MR activation in PASMC is also responsible for the perivascular inflammation in PH (47), likely through the production of a paracrine factor that enhances monocyte chemotaxis (48). Pharmacological MR blockade exerts protective effects against pulmonary vascular remodeling with decreased PASMC proliferation and reduced inflammatory cell infiltration (43). Likewise, a lower degree of perivascular lung inflammation in response to hypoxia-sugen was observed in mice with SMC-specific deletion of MR compared to wild type (47). In addition, aldosterone may exert adverse effects on PASMCs by promoting cell senescence as aldosterone has been shown as one the strong pro-senescent factors in the pathogenesis of cardiovascular diseases (49), which also play a crucial role in the pathobiology of PH (50). However, the evidence directly linking aldosterone signaling with cellular senescence in the development of PH is still lacking and should focus of the future investigation. Taken together, the pathologic effects of aldosterone-MR signaling in PASMCs are mainly driven due to their effects to promote cell proliferation, apoptosis resistance, ECM synthesis, and perivascular inflammation (Figure 2).

## Pharmacological targeting of MR in PH animal models

Several studies have been conducted to evaluate the potential benefits of MRAs to prevent or reverse pulmonary vascular remodeling in animal models of PH. Those experimental studies have employed MRAs spironolactone, eplerenone, or finerenone in several models of PH (Tables 1, 2). The overall finding of those studies is that MRAs can prevent and partially reverse pulmonary vascular remodeling and improve pulmonary hemodynamics in PH (43, 47, 51–53) (Tables 1, 2). Preventive application MRAs attenuated the development of adverse pulmonary vascular remodeling as assesses by attenuated PA muscularization and wall thickening along with decreased PA pressure (27, 44, 46, 47, 51, 52, 54) (Table 1). Initiation of MRA therapy after the disease phenotype had established was able to decrease PA muscularization and wall thickening and improve pulmonary hemodynamics (26, 43, 44, 46, 53) (Table 2). This applied both to steroidal as well as the novel non-steroidal MRA finerenone (43). In most of these studies, both preventive and therapeutic applications of MRAs could improve RV dysfunction and remodeling induced by monocrotaline, hypoxia, or hypoxia-sugen (43). It has been questioned whether improved RV function was a direct effect of MRAs on cardiac cells or indirectly mediated by lowered RV afterload.

In one recent study, MRAs initiated after the onset of significant RV failure in the hypoxia-sugen rat PH model led to modest, but consistent beneficial effects on cardiac function and remodeling (53). Specifically, MRI imaging of the heart in hypoxia-sugen rats, revealed that MRAs improved cardiac index, the RVEDV/LVEDV ratio, and the degree of septal displacement although no significant reductions in either PA pressure or vessel remodeling were observed (53). The cardioprotective effect of MRAs in this study may be related at least in part to the attenuated pro-inflammatory gene expression in the RV (53), which is considered as a crucial mediator of adverse RV remodeling in response to pressure overload (55). In contrast to that, application of the MRA eplerenone after 1-week of PAB surgery in mice did not improve RV function and remodeling at 3 weeks (51). Similarly, in the rat PAB model of RV remodeling, preventive application of an Ang-II receptor blocker plus eplerenone for 11 weeks also did improve RV function (56).

Taken together, these findings suggest that MR blockade can exert beneficial effects on pulmonary vascular remodeling and subsequent RV failure when applied preventive or in established mild-to-moderate PH. In more advanced pulmonary vascular remodeling or in the setting of fixed RV pressure overload, their therapeutic potential seems to be limited.

## Genetic manipulation of MR in PH animal models

During the past 10 years a series of experimental studies using transgenic mouse lines with cell type-specific targeting of the MR in the cardiovascular system have unraveled distinct roles for MR in SMCs, ECs, cardiomyocytes, and monocytes/macrophages. As a result of these studies, the specific contribution of in MR different cell types to hypertension, heart failure, or post-myocardial infarction remodeling could be defined (9, 10). A recent study has demonstrated that transgenic mice ubiquitously overexpressing human MR (hMR^+^ mice) spontaneously develop PH, characterized by increased RV systolic pressures, RV hypertrophy, and remodeling of small pulmonary vessels and a 2-fold increase in the percentage of proliferating PASMCs compared with their wall thickness (WT) littermates (43). This adds to an earlier study reporting that hMR^+^ mice develop moderate dilated cardiomyopathy without cardiac fibrosis, with normal blood pressure, tachycardia, and a high occurrence of arrhythmia (57). Given the many parallels in the pathophysiology of cardiovascular disease, it seemed plausible that MR in these cells might also be involved in the development of PH (Table 3). Indeed, while mice with EC-specific MR deletion were protected from pulmonary vascular remodeling in response to hypoxia in a similar manner as seen with eplerenone treatment, transgenic mouse lines with specific MR deletion in SMCs, fibroblast, or myeloid cells displayed a similar degree of PH as observed in WT mice (52). These findings indicate that the beneficial effects of MRAs on PH may be mainly mediated through the blockade of MR in ECs with indirect effects on PASMCs. It is important to note that this finding could not be reproduced in another study using the hypoxia-sugen model (47). EC-specific MR deletion has been shown to exert benefits on the RV in the hypoxia-sugen mouse model by regulating RV E-selectin and collagen III expression and attenuating RV perivascular fibrosis but did not improve PH (47). This suggests that the protective effects of MR deletion in PAECs on the pulmonary vasculature may involve VEGF signaling (as this remains disturbed in the hypoxia-sugen model due to inhibition of VEGF receptor 2 by SU5416, independent of the MR). In line with this, we had reported before that MR activation is able to counterregulate VEGF signaling in cultured endothelial cells (58). SMC-MR deletion did not improve PH and RV hypertrophy in both, hypoxia and hypoxia-sugen models of PH, compared to wild type mice (47, 52), although MR deficient mice displayed attenuated degree of lung perivascular inflammation (47). This finding suggests that PASMC-MR, activated *in vivo* in PH, contributes to the recruitment of inflammatory cells in the lung perivascular area. This most likely occurs through a yet to be defined paracrine factor that promotes chemotaxis of inflammatory cells. However, it is surprising that such anti-inflammatory benefits of SMC-MR deletion did not translate into the improved pulmonary hemodynamics and vascular remodeling. Taken together, these studies using transgenic mouse lines revealed that MR overexpression can cause the development of spontaneous PH while MR deletion can prevent the development of PH in response to hypoxia at least in part due to EC-specific actions.

**TABLE 3:** Summary of studies evaluating the effects of genetic manipulation of mineralocorticoid receptor (MR) on the pulmonary vasculature and right ventricle in rodent models.

Cell type	PH model	Genetic model	Pulmonary hemodynamics		Vascular remodeling (histo)	RV remodeling (echo)	RV remodeling (ex-vivo, histo)	RV function (echo)	References
			**Invasive**	**non-invasive**					
SMC MR deletion	Hypoxia mice (6 weeks)	Myh11^MerCreMer^–MR^fl/fl^	NA	↔ PAAT/PAET	NA	NA	NA	↔ TAPSE	(52)
Macrophage MR deletion	Hypoxia mice (6 weeks)	LysM^Cre^–MR^fl/fl^	NA	↔ PAAT/PAET	NA	NA	NA	↔ TAPSE	(52)
FB MR deletion	Hypoxia mice (6 weeks)	Tcf21^CreERT^–MR^fl/fl^	NA	↔ PAAT/PAET	NA	NA	NA	↔ TAPSE	(52)
EC MR deletion	Hypoxia mice (6 weeks)	Cdh5^CreERT^–MR^fl/fl^	NA	↑ PAAT/PAET	↓ PA thickness	↓ RVID/LVID	↓ RV myocyte	↑ TAPSE	(52)
SMC MR deletion	Hypoxia-sugen mice (4 weeks)	Acta2^Cre^–MR^fl/fl^	↔ RVSP	NA	↔ PA muscularization	NA	↔ RV/(LV+S), ↔ RV myocyte, ↔ RV perivascular fibrosis, ↔ RV interstitial fibrosis	NA	(47)
EC MR deletion	Hypoxia-sugen mice (4 weeks)	Cdh5^CreERT^–MR^fl/fl^	↔ RVSP	NA	↔ PA muscularization	NA	↔ RV/(LV+S), ↔ RV myocyte, ↓ RV perivascular fibrosis, ↔ RV interstitial fibrosis	NA	(47)
Global MR overexpression	hMR expressing mice	P1 promoter into the B6D2F1 mouse strain	↑ RVSP	↔ PAAT/PAET	↑ PA muscularization, ↑ PA wall thickness	NA	↑ RV/(LV+S)	NA	(43)

EC, endothelial cells; FB, fibroblasts; SMCs, smooth muscle cells; RVSP, right ventricular systolic pressure; BW, body weight; RV, right ventricle; LV, left ventricle; S, septum; PA, pulmonary artery; TAPSE, tricuspid annular plane systolic excursion; RVID, right ventricular diameter at end-diastole; LVID, left ventricular internal diameter. NA, not applicable. Parameter ↑ increased, ↓ decreased, or remains ↔ unchanged compared to wild type control.

## Clinical application of MR antagonists in PH patients

Above discussed preclinical studies and the established utility of MRAs for the management of heart failure have led to several clinical studies evaluating the potential benefit of MRAs in patients with PH (59). For example, a retrospective analysis of spironolactone use in PAH patients in ARIES-1 and ARIES-2 trials showed a trend toward improved 6-min walking distance (6-MWD) and circulating B-type natriuretic peptide (BNP) levels with the combination of spironolactone and ambrisentan (ET-A antagonist) and compared to ambrisentan alone (22). Interestingly, spironolactone use was associated with more potent decrease in circulating inflammatory markers compared to PAH-specific therapies (40). Another retrospective study revealed that MRA use indicates disease severity in PH patients (60). This may be the result of prescribing MRAs only for those PH patients with more severe conditions. It was documented that a combination of spironolactone and hydrochlorothiazide almost completely reversed PH and RV dysfunction within 3 months of treatment initiation in a preterm infant with bronchopulmonary dysplasia (BPD) with associated severe PH and RV failure (61). Cumulatively, the results of these clinical studies suggest that MRAs may be beneficial for PAH patients, and their use is associated with a worse clinical condition, likely due to a delayed initiation of MRAs. In contrast, two recent studies indicated that MRA use was associated with increased mortality in patients with PH (60, 62). In one of these studies, the association of MRA use with decreased survival was not evident after adjustment for disease severity, suggesting that MRAs were prescribed preferentially for those PH patients with more advanced disease condition (62). Another trial showed that spironolactone did not change tissue fibrosis biomarker levels as the primary end-point nor did it improve clinical outcomes, although spironolactone was well-tolerated and did not lead to significant adverse events in PAH patients (63). Of note, it was possible to analyse the effects of MRAs on PH patients in above discussed retrospective studies because MRAs are prescribed for PH patients with fluid overload. Whether MRAs improve outcomes of patients with PH due to left heart disease is unknown. The TOPCAT trial failed to demonstrate a benefit of spironolactone in patients with HFpEF, 36% of them showing PH (64, 65). However, the study had substantial methodological problems and must be interpreted with caution (66). Currently, there are two further prospective, randomized placebo-controlled trials are ongoing. The STAR-HF trial, assessing the ability of spironolactone to reduce RV ventricular wall stress (NCT03344159). Another study is evaluating the effect of spironolactone on exercise capacity, RV function, inflammatory markers, and potential side effects in PAH patients (NCT01712620). The results of these trials are expected to determine whether MR blockade is beneficial in managing PAH patients.

## Summary

Mineralocorticoid receptor is an important and highly versatile transcription factor that regulates various key signaling pathways in the pathogenesis of PH. MR activation in PAECs promotes aberrant redox signaling through augmented expression of pro-oxidant enzymes, increased ROS production, and reduced NO bioavailability, resulting in PAEC senescence and apoptosis. In addition, MR activation drives pro-inflammatory and pro-fibrotic phenotypes of PAECs. In PASMCs, MR activation causes cell hyperproliferation and excessive ECM synthesis and perivascular inflammation. Cumulatively, these effects of MR activation in pulmonary vascular cells promote PH development and progression. Abundant evidence from preclinical studies demonstrates the therapeutic promise of MR blockade to prevent and reverse many pathobiological features underlying PH. Considering that MRAs are available as approved treatment for left heart failure, repurposing those agents for the treatment of PH patients appears a promising strategy. Indeed, based on the available evidence on the regulation of aldosterone in PH patients and the results of retrospective clinical studies, subpopulations of PAH patients can be identified that may benefit from MRA treatment. Experimental studies may help to identify suitable biomarkers to closer define patients that are responsive to MRA treatment. However, data from a prospective randomized trial with MRAs in addition to established PH therapy is warranted to make definitive conclusions about the efficacy of MR blockade in the management of PH.

## Challenges and future directions

The use of animal models has proven to be a valuable tool in revealing the molecular mechanisms of PH and to test potential new therapeutic approaches. The available data from experimental studies have provided key insights into the role of MR signaling in the pathogenesis of PH and its potential as a therapeutic target. They indicate that increased MR activity leads to pulmonary vascular remodeling, ultimately resulting in the development of PH. MR activation modulates complex signaling pathways during PH pathogenesis, as a result of its cell-, tissue-, and organ-specific effects. In this regard, these multitude roles of MR signaling may also pose many challenges in the field of research because it makes it difficult to interpret and generalize the results obtained. A common problem encountered during preclinical research is the controversial results across available studies. For example, the effects of MRAs tested in rodent models of PH may differ substantially between studies. It is likely that the different methodological approaches employed in those studies account for the majority of controversial results and heterogeneous conclusions. Therefore, it is important to consider the following points in interpreting the results of already available studies or in designing new studies: (1) choosing the model that fits best with the PH class and severity of disease of the target patient collective (for example, hypoxia model leading to moderate PH, while hypoxia-sugen causes more severe PH phenotypes and interferes with specific signaling pathways); (2) whether studies include animals of both sexes (to take into account for the sex-specific features of MR signaling); (3) phenotyping the disease severity in a rodent model with state-of-the-art imaging methods such as echocardiography, MRI, and cardiac catheterization (for example MRI can help to detect subtle cardiac changes of MRAs more precisely, which may be missed by other imaging modality); (4) whether doses, durations, and routes of administration of MRAs are comparable across studies.

Majority of available studies evaluating the effects of MRAs in PH model, used only male animals. This makes it challenging to extrapolate the results obtained from male animals to their female counterparts due to several sex-specific differences in the cardiovascular physiology. For example, sex differences play an important role not only in the development of PH (67), but also in responses to MRAs (34, 68) and in vascular role of the MR (69, 70). A recent study demonstrated that increased aldosterone production in response to physiologic and pathophysiological stimuli, increased EC MR expression and increased susceptibility to aldosterone-induced EC dysfunction in females compared with males (71). In addition, there is evidence to suggest that therapeutic responses to MRAs may be greater in females compared to males (72, 73). Cumulatively, the results of these studies dictate that it is crucial to consider the sex-specific features in MR research in PH. Although, cell-specific roles of MR in PH models have been studied in major vascular cells including SMC, ECs, fibroblasts, and macrophages, the contribution of other cell type MRs in PH pathogenesis cannot be ruled out. For example, the MR in cardiomyocytes or T cells may also play a role in the pathogenesis of PH. In addition, differing dosages and routes of administration of MRAs may also account for some of the conflicting results obtained from the experimental studies. For example, MRAs were administered with chow (26, 27, 51, 52), from a subcutaneous continuously releasing pellet (44, 47), or with a drinking water (26, 27, 46, 54). Similarly, the dosages of MRAs differed by up to factor three between studies (51–53). Whether there is a dose-dependent effect of MRAs in PH has not been systematically assessed yet. In the end, well-designed prospective clinical trials will be needed to properly assess a potential benefit of MRAs in PH. Considering the insights and challenges outlined above may help us to design future clinical studies that evaluate the effects of (1) different MRA compounds in (2) patients from different groups of PH at (3) different stages of disease.

## Author contributions

AM and AL conceived, drafted, and revised manuscript and figures. Both authors contributed to the article and approved the submitted version.
